# The Story of the Insane from Year to Year

**Published:** 1892-04-02

**Authors:** 


### The Story of the Insane from Year to Year.

(Fourth Series.)

### SUSSEX COUNTY ASYLUM, HAYWARD'S HEATH.

The total number of admissions and re-admissions during the year was 289, and there were 844 patients in residence at the end of the year, against 801 in the previous year. The recoveries were 77, and the deaths 103, the former being at the rate of 23·2 per cent. on the admissions, and the latter at 11·2 per cent. on the average number of residents. In 54 of the admissions the insanity was caused by hereditary influence, and in 21 it was due to drink. Phthisis accounts for 21 of the deaths; bronchitis, pneumonia, and pleuro-pneumonia, collectively, for 10, and 12 were caused by progressive paralysis. Dr. Saunders says much of the new Lunacy Act "is complicated in machinery and somewhat contradictory in terms," and he especially refers to the re-certification of patients, which is certainly one of the most extraordinary provisions of an extraordinary Act, and which, we believe, caused in some asylums much commotion and discontent among the patients. The visitors say the condition of the asylum "reflects very creditably on Dr. Saunders and his staff;" and they urge on the County Council the necessity of providing further accommodation for the pauper lunatics of the county. Nearly one hundred patients are boarded out in other asylums at a considerable increase of cost to the ratepayers, and causing much annoyance to the friends of these patients, who are for the most part unable to travel long distances to see their sick relatives. This lack of room in Sussex proves once more what we have often expressed in these columns, that when a county does not anticipate its wants in asylum matters, it always lags behind these wants—often far behind. There is a balance of £530 in favour of the farm. Rent of land is charged and paid labour; but we see nothing put down for interest on capital nor patients' labour; and he will be a lucky farmer who can get elevenpence a gallon for milk, and ninepence a pound for mutton. The weekly rate of maintenance stands at 9·5¾.

### WEST RIDING ASYLUM, MENSTON.

During the year 1890 the numbers in this asylum went up from 486 to 679. The admissions were 405, the recoveries 119, and the deaths 69. Calculated in the usual way, the recoveries stand at 29·38 per cent., and the deaths 11·75. Drink was the assigned cause of the disease in 79 cases, and heredity in 105. Seven of the deaths were owing to phthisis. Dr. MacDowall finds, what most other superintendents have found before, that the applications for admission to the County Asylum increases year by year. He says: "The great increase in the number of private patients shows how greatly accommodation for this class of case is required in public asylums. At present it is impossible to separate such cases from patients sent by the various unions, and this, in many instances at least, is undoubtedly unfortunate, and acts prejudicially on the patients. A building with smaller rooms, and offering greater facilities for classification, would greatly facilitate the treatment of such cases, and would remove the greatest objection which the friends have to the existing arrangement." The new Lunacy Bill gives the Boards of Visitors of our County Asylums the power to build for private patients; and we hope that in his next report Dr. MacDowall will be able to tell us the Act is being carried out at Menston, and a good example set to other counties. Out of the 405 admissions, there were 27 congenital cases, 24 epileptics, 51 paralytics, 56 chronic cases, 20 dements, and seven cases of brain disease, making a total of 205 patients whose cases Dr. MacDowall says are "utterly hopeless." Has he never heard that a small acute hospital in the grounds at Menston would enable him to cure over 60 per cent. of his cases? Has he never reflected how surely, with that hospital at his elbow, the secondary dements would brighten up, the general paralytics would cease stammering, the epileptics give up quarrelling, and the melancholics would no longer brood over their sorrows? But perhaps Dr. MacDowall does not believe it all. The farm accounts show a balance of £150 in favour of the farm. Rent of land is charged in the accounts, but not patients' labour nor interest on capital. The weekly cost per head is 8s. 10d.

### HULL BOROUGH ASYLUM.

There were 321 patients in this asylum at the close of the year, being an increase of eight on the previous twelve months. The admissions were 98, the recoveries 31, and the deaths 46. The recoveries yield a percentage of 31·63, and the deaths 14·37. Twenty-three of the admissions had their insanity caused by drink, and in the same number hereditary influence was the ascertained cause. Dr. Merson's report is extremely clear and able, and shows how carefully he examines, and how accurately he gauges everything connected with his asylum. He tells us that nearly 70 per cent. of the admissions were unlikely to recover. This only leaves 30 per cent. of curable cases; but, then, Dr. Merson is unprovided with an acute hospital, which would at one fell swoop double his curable cases. Perhaps the Hull Asylum is too small to provide a hospital of this kind; but it would be easy to call one ward the "hospital ward," and fit up a single room with a microscope and half-a-dozen test-tubes. If Dr. Merson were to do this, he would be surprised, and for the matter of that so should we, at the sudden rise in his percentage of cures. The Commissioners call attention to the high death-rate, which they believe (without any exceptional disorder) "to be unprecedented in asylum annals." Dr. Merson replies "that it is very unsafe to draw any conclusion from the mere figures, without giving due weight to the special circumstances of each case. A low death-rate is not necessarily, or even generally, an indication of a satisfactory hygienic condition, nor a high death-rate of the opposite. More often a low death-rate simply signifies a large chronic population with few admissions, and those of a very favourable kind. A high death-rate, on the other hand, may, and often does, mean a small number of chronic cases, frequent changes among the population, and an unfavourable type of admissions." This is really very well put. The visitors say they "have great pleasure in again expressing their entire satisfaction with the manner the patients have been looked after, and the interest of the institution has been attended to by the Medical Superintendent." What a profitable occupation is farming! These accounts show a profit of £372.

### PORTSMOUTH BOROUGH ASYLUM.

On December 31st, 1890, there were 528 patients in residence. The admissions were 115, the deaths 44. The recoveries were 46. Calculated on the admissions, the recoveries stand at 38·5 per cent., and the deaths, calculated on the average number resident, yield a percentage of 6·7. The former is a trifle under the average, and the latter very much under it. Drink was the cause in ten of the cases admitted, and hereditary taint in seven. The latter is probably lower than would be disclosed by a strict investigation, for we find the cause not stated in 23, and "previous attack" is given as the cause in 13. It cannot be doubted that many of these 36 cases owed their insanity to drink and heredity. Consumption killed 13 patients, and general paralysis is credited with the same number. *Post mortem* examinations were made in 38 instances. An inquest was held on the body of a man who died of brain softening and Bright's disease, but in whom three ribs were found broken. The ribs were thin and brittle, and easily fractured. This brittleness has often been found in the ribs of the insane, and in some it exists to such an extent that very little pressure will fracture the bones. The fact certainly explains some of those accidents occurring in asylums where attendants have been falsely accused of rough usage. In this instance the jury exonerated the officials from all blame. The weekly cost of maintenance is a fraction under 9s. 10d. per head.

